# Artificial Intelligence-Based Drone System for Multiclass Plant Disease Detection Using an Improved Efficient Convolutional Neural Network

**DOI:** 10.3389/fpls.2022.808380

**Published:** 2022-06-09

**Authors:** Waleed Albattah, Ali Javed, Marriam Nawaz, Momina Masood, Saleh Albahli

**Affiliations:** ^1^Department of Information Technology, College of Computer, Qassim University, Buraydah, Saudi Arabia; ^2^Department of Computer Science, University of Engineering and Technology Taxila, Taxila, Pakistan

**Keywords:** deep learning, plant disease, CNN, agriculture, classification, EfficientNetV2

## Abstract

The role of agricultural development is very important in the economy of a country. However, the occurrence of several plant diseases is a major hindrance to the growth rate and quality of crops. The exact determination and categorization of crop leaf diseases is a complex and time-required activity due to the occurrence of low contrast information in the input samples. Moreover, the alterations in the size, location, structure of crop diseased portion, and existence of noise and blurriness effect in the input images further complicate the classification task. To solve the problems of existing techniques, a robust drone-based deep learning approach is proposed. More specifically, we have introduced an improved EfficientNetV2-B4 with additional added dense layers at the end of the architecture. The customized EfficientNetV2-B4 calculates the deep key points and classifies them in their related classes by utilizing an end-to-end training architecture. For performance evaluation, a standard dataset, namely, the PlantVillage Kaggle along with the samples captured using a drone is used which is complicated in the aspect of varying image samples with diverse image capturing conditions. We attained the average precision, recall, and accuracy values of 99.63, 99.93, and 99.99%, respectively. The obtained results confirm the robustness of our approach in comparison to other recent techniques and also show less time complexity.

## Introduction

Agriculture played a vital role in the domestication of today’s major food crops and livestock thousands of years ago. Food insecurity is one of the serious worldwide concerns that humanity is facing today, caused by plant diseases (Ristaino et al., 2021). Plant diseases cause crop damage and majorly affect the overall crop production yield, which leads to food shortages (Strange and Scott, 2005). According to Food and Agriculture Organization estimates, plant pests and diseases are responsible for up to 40% of global agriculture production loss (Fenu and Malloci, 2021). This may result in disastrous consequences, such as millions of people not having enough food and severely harming the agriculture sector. Moreover, smallholder farmers provide more than 80% of agricultural production in developing countries, which is their main source of livelihood. Furthermore, the majority of poor people approximately 50% reside in smallholder agricultural families, making smallholder farmers particularly vulnerable to pathogen-related food supply disruptions (Mohanty et al., 2016). Therefore, finding new ways to identify plant diseases can significantly improve the food yield and turn the losses into profit.

The management of large-scale agricultural production necessitates a variety of timely actions, such as keeping an eye out for diseases and which limits them to unwanted items. The most common causes of plant disease are insect pests, bacteria, viruses, algae, and fungi. Certain plant diseases have no visual indications; therefore, advanced analytical methods are used in these cases (Sankaran et al., 2010; Dinh et al., 2020). However, most of the infected plants have visible signs, and an experienced plant pathologist identifies the disease by examining infected plant leaves using an optical microscope. An accurate plant disease diagnosis requires good observation skills and knowledge to recognize precise symptoms of a certain disease. This manual plant disease identification process is time-consuming and dependent on the availability of experienced plant pathologists. Moreover, continual plant monitoring is required, which is highly expensive when dealing with large farms. Furthermore, the excessive variety of plants and variations in symptoms over time due to climate changes even the experienced pathologist may unable to accurately identify certain diseases and may take a long time. For sustainable and correct agriculture, as well as to avoid unnecessary waste of financial and other resources, timely and precise identification of plant diseases is critical.

In recent years, due to the advancements in technology, image-based automated process control systems are introduced that can automatically identify the disease plants and offer valuable insight to agronomists (Singh et al., 2020). Automatic detection techniques assist farmers in improving crop quality while also reducing disease occurrence through early identification, timely, and appropriate treatment. Image processing is used for measuring the affected area of disease and to determine the difference in the color of the affected area. Initially, machine learning (ML)-based models were proposed for the identification and classification of plant diseases. The methods such as support vector machine (SVM) (Elangovan and Nalini, 2017), decision tree (DT) (Rokach and Maimon, 2005), random forest (RF) (Ramesh et al., 2018), and K-nearest neighbors (KNN) (Liao and Vemuri, 2002) have been employed for early and accurate detection of crop plant diseases. The ML-based techniques are simpler to deploy and do not require huge training data; however, they are slow due to complex preprocessing and dependent on the knowledge of experienced human specialists for extraction and the selection of suitable features required to perform the classification (Dargan et al., 2020). Moreover, selecting a large feature set increases the computing complexity, while using a small feature set degrades the identification performance. Therefore, the detection efficacy of these approaches depends on the quality and representation of extracted features and is susceptible to errors when working with a large amount of data. Thus, ML-based techniques have limited accuracy for automated plant disease identification.

Now, deep learning (DL)-based approaches are extensively being applied in different fields including agriculture as well (Zhang et al., 2019; Liu and Wang, 2021). These techniques automatically compute discriminative features directly from the input samples, thus avoiding complicated image preprocessing and reducing the memory footprint. The Convolutional Neural Network (CNN) is a well-known DL model that showed effective performance in pattern recognition and is widely employed for early plant leaf disease identification. In recent studies (Lu et al., 2017; Ahila Priyadharshini et al., 2019; Agarwal et al., 2020), CNN is primarily used for crop plant disease identification and classification. These approaches have shown promising results in crop-related classification tasks due to effective feature representation. The mature CNN architectures in computer vision such as AlexNet (Rangarajan et al., 2018), GoogleNet (Mohanty et al., 2016), VGGNet (Chen et al., 2020a), ResNet (Richey et al., 2020), EfficientNet (Atila et al., 2021; Chowdhury et al., 2021), mobileNet (Bi et al., 2020), and Densenet (Waheed et al., 2020) are extensively used in existing plant disease categorization methods. Some studies (Wen et al., 2020; Atila et al., 2021) have designed custom network architectures to tackle real-world scenarios such as occlusion, low light, and different climate environments. At present, object detection algorithms based on DL are constantly being developed and adopted for plant disease localization and classification (Zhou et al., 2019; Zhang et al., 2020). These methods determine the precise location and class of the disease; however, for a real complex natural environment, the performance degrades. Despite recent progress, there is still a need for improvement in the application of DL architectures, particularly novel DL architectures for crop plant disease classification in terms of generalization robustness and identification accuracy. Moreover, the requirement for efficient models with fewer training parameters and faster training speed without compromising the performance is unavoidable.

The accurate identification of several plant diseases is still challenging because the diseased spots have varying appearances, such as size, shape, hues, and position. Moreover, the presence of background noise, intra-class differences at different growth stages, and multiple tiny and dense diseased spots on the same leaf affect the diagnosis of plant leaf diseases. Furthermore, variations in illumination and brightness conditions during the image acquisition process of leaves contribute to the unsatisfactory detection results of computer-aided design (CAD) solutions. To deal with the aforementioned challenges, this study proposes an efficient and effective drone-based method, namely, improved EfficientNetV2 for developing crop-disease classification tools. The custom EfficientNetV2 model computes the deep features and classifies them in their respective class using an end-to-end training architecture. The presented framework, namely, the improved EfficientNetV2 is better than the original network in terms of detection accuracy, time, and number of model parameters. Moreover, the proposed method is more robust to unseen examples as well due to the inclusion of dense layers. The main contributions of our study are as follows:

• Fine-tuned an image classification approach, namely, improved EfficientNetV2 for the real-time plant disease classification which improved the classification performance.
• We proposed a cost-effective framework to enhance the plant disease recognition accuracy while minimizing the training and testing time.
• Extensive performance analysis has been conducted using a standard database, namely, PlantVillage, and a detailed comparison has been performed against several recent approaches to demonstrate the efficacy of the proposed method.
• The introduced approach is capable of effectively classifying the plant disease under varying challenging conditions such as the presence of blurring, noise, and variations in color, size, and position of the infected regions.

The rest of the study is organized as follows: the “Related work” section presents the overview of the related work and its limitations. The “Materials and methods” section presents the proposed plant leaf disease diagnoses method. The “Experiment and results” section presents the experimental details, analysis, and discussion on obtained results. Finally, the “Conclusion” section summarizes the study.

## Related Work

In this study, a critical investigation of existing research for the detection and classification of various plant diseases is performed. Present literature for plant leaf diseased region categorization is divided into two types, namely, ML-based methods or DL-based approaches.

Kaur (2021) proposed an ML-based approach for plant disease classification. Several feature extraction algorithms, namely, Local Binary Pattern (LBP), Gray Level Co-Occurrence Matrix (GLCM), Shift-Invariant Feature Transform (SIFT), and Gabor were applied for feature extraction from the input images. Then, the ML classifiers, namely, SVM, KNN, Artificial Neural Network (ANN), and RF were trained to accomplish the plant disease categorization task. The study by Kaur (2021) attains the best result for Gabor features with the classification accuracy of 90.23%; however, performance needs further improvements. Shrivastava and Pradhan (2021) proposed a solution for the detection and classification of different plant diseases. Initially, the 14 color spaces were used to extract the 172 key points from the suspected samples. Then, the calculated key points were used for the training of SVM. The approach by Shrivastava and Pradhan (2021) demonstrates improved plant leaf diseased region categorization performance with an accuracy of 94.68%; however, this approach is not robust to samples with huge distortions. Le et al. (2020) presented an approach to locate and categorize the crops and weed-based diseases. In the first step, the noise from the suspected samples was removed by employing the morphological opening and closing operation. In the next step, a custom model, namely, the filtered LBP approach along with contour mask and coefficient k (k-FLBPCM) was introduced to extract the key points from the enhanced image. Finally, the computed key points were employed for the SVM training to achieve the diseased leaf region categorization. The technique by Le et al. (2020) shows improved plant disease recognition power with an accuracy of 98.63%; however, this approach is not robust for the image containing perspective distortions. Ahmad et al. (2020) presented a method to identify and recognize the affected areas of several plant leaves. Initially, Directional Local Quinary Patterns (DLQPs) were used for feature extraction from the input images. The calculated key points were utilized for training the SVM classifier to classify the crop leaf diseases. This method (Ahmad et al., 2020) works well for plant disease classification with the classification accuracy of 96.50%; however, detection performance can be further enhanced using the shape and color-based information of the suspected images. Another approach to detect and classify tea plant leaf diseases was presented in the study by Sun et al. (2019). Initially, the Simple Linear Iterative Cluster (SLIC) was used to divide the input image into several blocks, from which the features were computed *via* the Harris method. Then, the convex hull method was applied to detect the fuzzy salient areas, and GLCM technique was utilized to calculate the feature vector. Finally, the extracted keypoints were used to train the SVM classifier. The framework (Sun et al., 2019) exhibits better crop leaf disease categorization with the accuracy of 98.50%; however, this method is economically complex. Pantazi et al. (2019) presented a method to locate and categorize several crop diseases. In the first step, the GrabCut approach was used over the suspected image to perform the sample segmentation. In the next step, the Hue, Saturation, and Value (HSV) transform was applied to the segmented image from which the features were computed using the LBP algorithm. Finally, the extracted key points were used to train the SVM. The study by Pantazi et al. (2019) is effective to plant leaf affected region categorization with the accuracy of 95%; however, its detection accuracy degrades over the noisy samples. Another ML-based technique was used by Oo and Htun (2018) to classify several crop diseases. Initially, the input samples were preprocessed by applying the histogram equalization (HE) approach to improve the visual information of images. In the next step, the K-means clustering method was applied to perform the image segmentation. Then, the GLCM along with the LBP descriptors were utilized for keypoint computation. Finally, the SVM algorithm was trained over the computed features for classifying the various crop leaf affected areas. The study by Oo and Htun (2018) exhibits enhanced crop leaf diseased region classification performance with the accuracy of 84.6%; however, evaluation is performed on a database with a small number of samples. Ramesh et al. (2018) introduced a technique to categorize the various abnormalities of crop leaves. The Histogram of Oriented Gradient (HOG) approach was applied for keypoint computation, which was employed for the RF classifier training. The method described by Liao and Vemuri (2002) and Ramesh et al. (2018) shows better crop leaf disease categorization with the accuracy of 70.14%; however, performance requires more enhancement. Kuricheti and Supriya (2019) proposed a solution to accomplish the classification task of categorizing turmeric leaf abnormalities. Initially, a preprocessing step was employed to improve the visual presentation of samples. Then the K-means method was used to the enhanced samples to accomplish the regional clustering. After this, the GLCM technique was used to extract the keypoints. Then, the calculated features were utilized for the SVM classifier to execute the diseased region categorization task. The approach by Kuricheti and Supriya (2019) demonstrates a better classification accuracy of 91%, however, exhibiting poor performance for image containing intense brightness changes.

Atila et al. (2021) proposed a DL-based method, namely, the EfficientNet framework for the identification and categorization of crop diseases. The performance of the model was evaluated over both the original and augmented dataset. The approach by Atila et al. (2021) performs well for plant disease classification with an accuracy of 99.97%, however, at the rate of higher computational complexity. Shah et al. (2021) proposed a solution for the automated classification of plant diseases by proposing a DL-based framework, namely, the Residual Teacher/Student (ResTS) model. The ResTS approach applied a CNN model for computing the deep features of an input sample. Then two classifiers, namely, the ResTeacher and ResStudent along with a decoder were applied to accomplish the categorization of various plant leaf abnormalities. The methodology presented by Shah et al. (2021) works well to perform the crop leaf disease categorization with the F1-score of 99.10%, however, unable to show robust performance in the presence of intense light variations. Another DL-based method, namely, ResNet50 was used by Sravan et al. (2021) for computing the deep features and classifying several plant diseases. The study by Sravan et al. (2021) is effective to perform the classification of several crop leaf affected portions with the accuracy of 99.26%, however, suffering from a high computational cost. Dwivedi et al. (2021) introduced a framework, namely, region-based CNN (RCNN) for localizing and classifying the grape plant diseases. Initially, ResNet18 was applied for computing the deep features which were later classified by the RCNN classifier. The approach by Dwivedi et al. (2021) shows improved results to categorize several diseases of the grape plant with an accuracy value of 99.93%, however, shows poor performance for real-world scenarios. Akshai and Anitha (2021) introduced several DL-based frameworks, namely, the VGG, ResNet, and DenseNet approaches for computing the deep features and classifying the plant diseases from the input samples. The method (Akshai and Anitha, 2021) attains the best performance for the DenseNet framework with the accuracy of 98.27%, however, at the expense of increased computational complexity. Another CNN-based approach, namely, Few-Shot Learning (FSL) was proposed by Argüeso et al. (2020) to identify and categorize the plant leaf affected portions. After performing the preprocessing step, the Inception V3 model was applied for computing the deep features from the input image. After the feature computation, a multiclass SVM classifier was used to train it over the key points to perform the classification task. The study by Argüeso et al. (2020) performs well to categorize the several diseases of plant leaves with an accuracy of 91.4%; however, evaluation results are discussed over a database of small size. Another CNN-based approach was presented by Agarwal et al. (2020) to detect and classify tomato crop disease. The approach (Agarwal et al., 2020) comprises 3 convolution layers together with max-pooling layers to compute the deep features from the suspected images and categorize them. This framework (Agarwal et al., 2020) shows robust tomato disease recognition performance with the classification score of 91.2%, however, suffers from the model over-fitting problem.

Richey et al. (2020) presented a lightweight approach to locate and recognize different diseases of the maize plant. The approach ResNet50 was used for computing the features from the samples under investigation which are then categorized into relevant groups. The approach by Richey et al. (2020) gives a low-cost framework for classifying the crop diseases with the classification accuracy of 99%; however, the work is less efficient to be employed on mobile phones due to limited power, execution, and space constraints. Another study employing the concept of transfer learning was introduced by Zhang et al. (2020) to categorize several tomato plant diseases. The study introduced a custom Faster-RCNN framework that employed a deep residual model to calculate the image features as an alternative to using the VGG16 network. Furthermore, the k-means clustering method was applied to cluster the localized regions. This study (Zhang et al., 2020) shows improved tomato crop leaf disease region categorization results with the mAP score of 98.54%, however, running from the extensive processing burden. Moreover, in the study by Batool et al. (2020), a methodology was presented to locate and categorize several tomato leaf-affected areas. First, the AlexNet model was applied to calculate the deep features from the input image that were utilized for training the KNN classifier. This study (Batool et al., 2020) demonstrates robust categorization performance with an accuracy of 76.1%; however, the KNN approach is a tedious and time-taking algorithm. Karthik et al. (2020) presented a method *via* using the concept of transfer learning to identify the affected regions of the leaves of the tomato crop. Moreover, in the study by Chowdhury et al. (2021), a ResNet network was used to calculate the key points from the suspected images. After extracting the representative set of image features, a CNN-based classifier was employed to perform the classification task. The study by Karthik et al. (2020) exhibits better crop leaf diseased region classification performance with an accuracy of 98%; however, the approach is economically expensive. Tm et al. (2018) proposed a DL technique for crop disease categorization. Initially, the suspected images were rescaled before employing them for further processing. Then, the LeNet approach was used to extract the representative set of keypoints and categorize the images into healthy and unhealthy classes. The method (Tm et al., 2018) gives a computationally efficient approach to classify the diseases of tomato crop with the classification accuracy of 95%, however, exhibits poor performance for images containing the noise.

We have reviewed several plant leaf disease classification techniques either using the conventional ML-based methods or DL-based approaches. However, there is a need for performance improvement both for the different plant leaf diseased region categorization and processing time complexities. The existing methods are employing either extensive preprocessing steps or are unable to perform well on distorted samples. Moreover, the methods are suffering from model over-fitting problems and do not achieve better performance over unseen samples. Therefore, the correct identification and classification of plant leaf disease are necessary to prevent crop damages which can further help the farmers to take timely precautionary measures.

## Materials and Methods

This section explains the dataset and methodology adopted for the identification of plant diseases. Moreover, the details of employed evaluation metrics are also discussed. For an input image, the ultimate aim is to automatically identify and determine the particular class of plant disease. Initially, the input samples comprising different species and diseases of the plant are collected from the publically available dataset and from the real-world environment *via* using the drone camera. These images are then fed to a customized EfficientNetV2 DL model for the computation of features and classification. We have presented an improved EfficientNetV2 model by introducing additional dense layers at the end of the framework which is the main contribution of our work. First, deep features are calculated using the deep layers, which are used to correctly classify the plant diseases of several types *via* using the Softmax classifier. The proposed CNN is explained in the “Proposed CNN” section.

### Dataset

**FIGURE 1:**
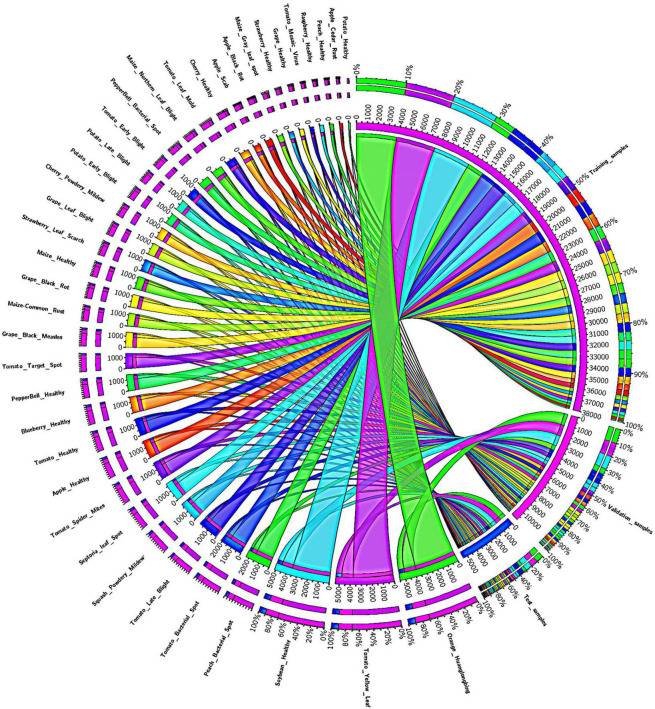
Class-wise detailed description of the PlantVillage dataset.

**FIGURE 2:**
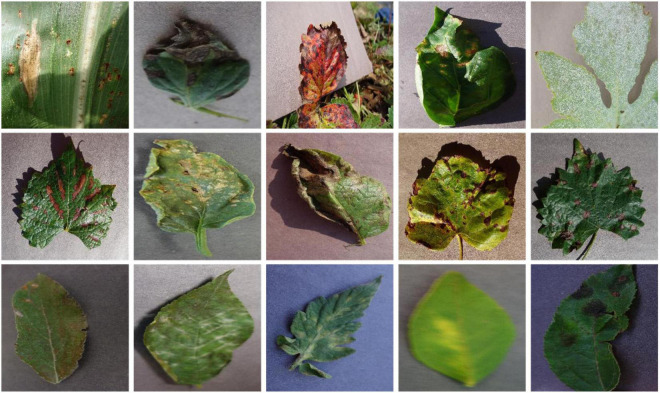
Sample images of PlantVillage dataset.

To evaluate the plant disease detection and classification performance of our approach, we have employed the PlantVillage database (Albattah et al., 2022). The PlantVillage dataset is a large and online accessible standard database of crop leaf disease classification, which is extensively explored by several techniques from the past for performance assessment. To check the classification accuracy of the presented method, we have designed several experiments on this dataset, which contains images from several types of plants and their diseases. More specifically, the PlantVillage dataset comprises 54,306 samples of 14 different species of plants and contains a total of 32 classes, of which 26 classes are from the diseased plants while the remaining 12 classes belong to healthy plants (Figure 1). The images from all the categories of plants containing Tomato, Strawberry, Grape, and Orange are taken from the PlantVillage database. The employed dataset is a diverse and challenging database for plant leaf affected region detection and categorization as it contains the samples which are varied in terms of plant leaf diseased portion size, color, and light, suffering from several image distortions such as the presence of noise, blurring, and color variations. A detailed description of the PlantVillage dataset is presented in Figure 1, and a few samples from the dataset are shown in Figure 2. Additionally, we have captured the real-time samples of a few species under natural lighting conditions to validate the proposed model. Approximately 30 colored samples were captured for a few available species. For this purpose, we used a quadcopter DJI Phantom 3 standard drone device. The specifications of the drone are Sensor: 1/2.3′′ CMOS, resolution: 4,000 × 3,000 pixels, and FOV: 94o. Multiple flights were used with an altitude between 5 and 6 m from the ground to capture the samples.

### Evaluation Parameters

Several standard evaluation metrics, namely, precision, recall, F1-score, and accuracy are used to check the categorization ability of our work in classifying the several categories of plant leaf diseases. We have measured the accuracy using the following equation:


(1) $$Accuracy=\frac{T_{p}+T_{n}}{T_{p}+F_{p}+T_{n}+F_{n}}$$

The following equations compute the precision, recall, and F1-score, respectively:


(2) $$Precision=\frac{T_{p}}{T_{p}+F_{p}}$$

(3) $$Recall=\frac{T_{p}}{T_{p}+F_{n}}$$

(4) $$F1=2\times \frac{Precision\times Recall}{Precision+Recall}$$

In this study, *T_p_*, *T_n_*, *F_p_*, and *F_n_* is demonstrating the true positive, true negative, false positive, and false-negative, respectively.

### Proposed Convolutional Neural Network

An effective feature representation is mandatory to attain accurate classification because the prediction performance of the classifier is directly associated with the quality of extracted features. As a result, a high-performance CNN must be used as the feature extractor to obtain discriminative features from images and generate compact feature vector representation. The CNN provides better image recognition when its neural network feature extraction becomes deeper (contains more layers) (Szegedy et al., 2015). However, the development of CNN architecture is dependent on the availability of computational resources, and the scaling occurs as the resources increase to obtain higher performance. To overcome the limitations of existing DL models, an efficient network having fewer parameters but higher classification accuracy is required (Ngugi et al., 2021). In this study, we proposed a lightweight, computationally efficient framework to increase the overall efficiency of the architecture for predicting plant leaf disease.

**FIGURE 3:**
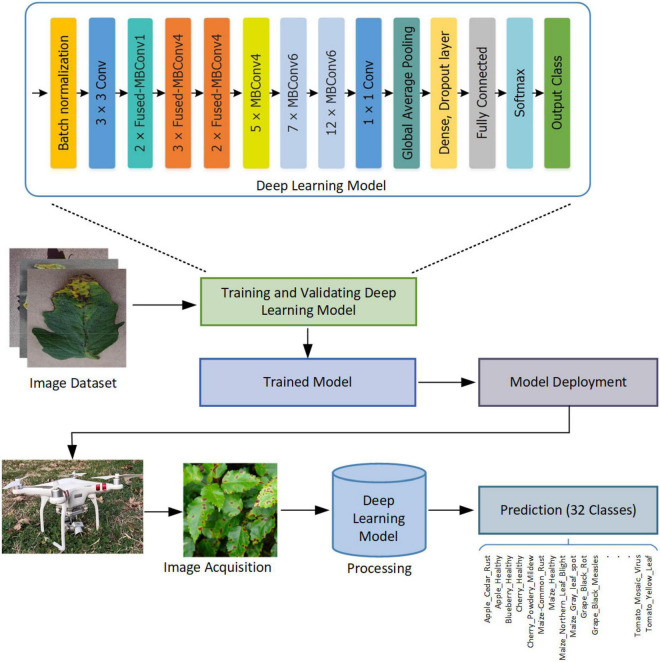
Architecture of the proposed model based on improved EfficientNetV2-B4.

Our architecture of CNN is based on the EfficientNetV2 model (Tan and Le, 2021). The EfficientNetV2 is an improved version of the EfficientNet CNN family (Tan and Le, 2019) and introduced to optimize available resources while maintaining high accuracies. EfficientNets architecture is built on a simple and efficient compound scaling technique that allows a baseline ConvNet to be scaled up to any resource restrictions while keeping the model efficiency. Thus, these models provide a good trade-off between computational complexity and optimal choice of network dimensions, i.e., depth of NN, width of convolutional layer, and size of input features. The EfficientNet models have fewer parameters and perform better in the classification task. As compared with previous CNN models such as AlexNet (Krizhevsky et al., 2012), GoogleNet (Szegedy et al., 2015), ResNet (Allen-Zhu and Li, 2019), DenseNet (Albahli et al., 2021), and MobileNet (Qin et al., 2018), the EfficientNet (Tan and Le, 2019) model achieved state-of-the-art performance in terms of accuracy and efficiency. Figure 3 shows the architecture of the proposed plant disease classification model.

**FIGURE 4:**
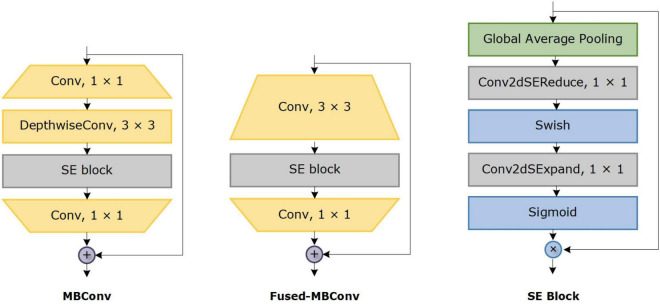
Structural details of MBConv4, Fused-MBConv4, and SE block.

The motivation to apply EfficiceinetV2 for plant disease classification is that it is lightweight architecture having faster inference time, training speed, and fewer parameters. EfficientNetV2 models employ neural architecture search (NAS) to optimize the model accuracy, size, and training speed simultaneously. In EfficientNetV2 architecture, Fused-MBConv blocks (Gupta and Akin, 2020) are added to improve the operational intensity and better utilize mobile or server accelerators. However, in EfficientNet architecture, only MBConv blocks (Sandler et al., 2018) are used as the basic building block, which includes depth-wise convolutions. The depth-wise convolutions have reduced arithmetic operations; however, they often cannot fully utilize the modern hardware accelerators. The EfficientNetV2 architecture extensively utilizes both the MBConv and Fused-MBConv blocks. In Fused-MBConv, the depth-wise 3 × 3 convolution and expansion 1 × 1 convolution in MBConv are replaced with regular 3 × 3 convolution layers (Figure 4). In EfficientNetV2, it was demonstrated that replacing certain MBConv blocks with Fused-MBConv blocks improves the model training speed and efficiency (Tan and Le, 2021).

For the classification of plant disease, we have improved the existing EfficientNetV2-B4 model and employed transfer-learning to provide a good trade-off between the accuracy and computational complexity. A detailed description of the proposed architecture is given in Table 1. The improvements in the existing architecture are shown in Table 1 in bold text. In our modified EfficientNetV2 model, a Batch normalization layer is added at the start of a network to standardize the inputs. The architecture comprises of Fused-MBConv block, and later layers use the MBConv blocks having 3 × 3 and 5 × 5 depth-wise convolutions with squeeze-and-excitation (SE) block (Hu et al., 2018) and swish activation. The MBConv block is an inverted residual block that uses an inverted residual connection along with the SE block to further enhance the performance. The SE block employs an attention mechanism to enhance feature representations that allow the model to prioritize infected regions of plant leaf through self-learning of weights. The architecture of the SE block is shown in Figure 4. The network uses the swish function (Ramachandran et al., 2017) as an activation function instead of ReLU because it discards values smaller than zero which may result in the loss of important information. While in comparison, the swish function permits the small negative weights to be transmitted through the network which is an important attribute to attain better performance for deep neural networks because such negative values are significant for computing patterns underlying the data. Therefore, the swish function assists the employed model to attain better classification results. Swish is defined as follows:


(5) Swish(x)=x×sigmoid(x)

**TABLE 1:** Details of blocks and layers used in the proposed model.

Block/Layer	Resolution	Channel
BatchNorm	**224 × 224**	**3**
Conv (3 × 3)	112 × 112	40
2 × Fused-MBConv1	112 × 112	16
3 × Fused-MBConv4	56 × 56	40
2 × Fused-MBConv4	28 × 28	56
5 × MBConv4	14 × 14	112
7 × MBConv6	14 × 14	136
12 × MBConv6	7 × 7	232
Conv (1 × 1)	7 × 7	232
Global average pooling	**1792**	
Dense	**128**	
Dropout	**128**	
Dense	**64**	
Dropout	**64**	
Dense	**32**	
Fully connected (FC)		
Softmax		

*Bold means the architectures are improved.*

In our model, only three fused-MBConv blocks are employed in the early layers because they contain a significant number of parameters for large values of *C* (the number of output channels) (Table 1). At the end of the CNN model, we added a global average pooling layer to reduce the overfitting by decreasing the overall number of parameters. A series of three inner dense layers with RELU activation functions and dropout layers have also been included. To prevent overfitting, a dropout rate of 30% was chosen at random. Finally, a dense layer consisting of 38 output units with a Softmax activation function was added to build the proposed automated plant disease detection system.

### Loss Function

The loss function is primarily used to evaluate the model’s efficacy. For a large amount of data, the system uses automated learning to identify the rules and make predictions. The loss function calculates the amount of variation between the actual and predicted values. The function is continually updated during the network training process until the best fitting result is obtained to decrease the error. When dealing with the classification problems, the soft-max layer employs the cross-entropy loss function (Su and Wang, 2020) that is extensively employed to assess the difference between the estimated and actual values. The following equation shows the definition of the cross-entropy loss function *L*:


(6) $$L=\frac{1}{n}\sum_{j=1}^{n}\text{log}\left(\frac{e^{s_{i}}}{\sum ke^{s_{j}}}\right)$$

where *n* is the number of neurons in the output layer and *s_i_* is the input vector.

## Experiment and Results

In this section, an in-depth demonstration of the acquired evaluation results for classifying several diseases of the plant leaves is given. Moreover, we have discussed the employed dataset along with the performance evaluation metrics. The entire framework is implemented in Python language and executed on an Nvidia GTX1070 GPU-based system, and model parameters for training are discussed in Table 2. For implementation, we have divided the employed dataset into 70–30% for the model training and testing, respectively.

**TABLE 2:** Description of framework hyper-parameters.

Network parameters	Value
Epochs	10
Learning rate score	0.001
Batch size	12
Optimizer	Stochastic gradient descent (SGD)

**FIGURE 5:**
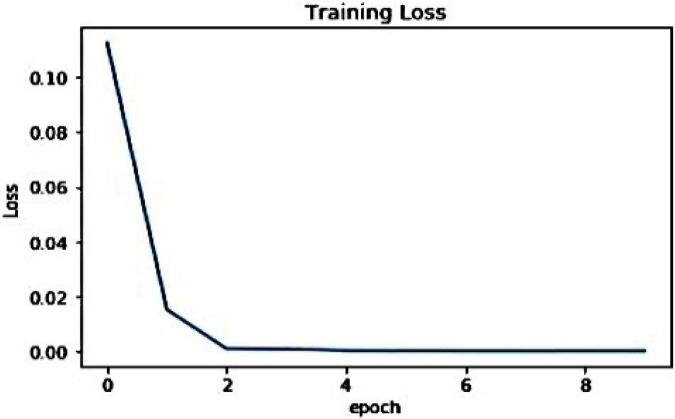
Visual representation of training loss graph.

**FIGURE 6:**
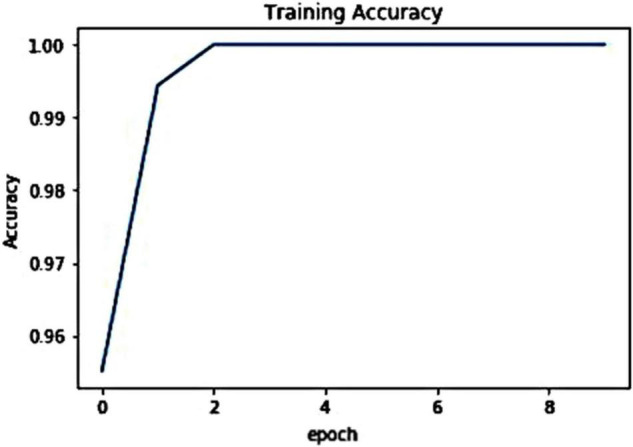
Visual representation of training accuracy graph.

To show the model behavior at training time, we have discussed both the train time plant disease classification accuracy and training loss as these parameters help to show the model behavior during the training procedure. For the presented approach, we have shown the visual representation of the loss graph in Figure 5. From Figure 5, we can clearly observe that the presented work acquired an optimal value of 0.0012 at the epoch number of 10, which is demonstrating the efficient learning of the proposed technique. Moreover, the improved ResNet50 model attains the highest validation accuracy of 100% as shown in Figure 6. The presented solution is robust to plant leaf disease classification due to its shallow network structure which permits better employment of obtained information by eliminating the redundant data. Due to such a framework structure, the custom EfficientNetV2 minimizes the total model parameters. As it contains a less deep framework structure with the power of better nominating the representative set of image deep features, which assists the improved EfficientNetV2 approach to enhance the classification performance while decreasing the computational cost as well. Such a setting of model architecture allows it to obtain the training convergence within 10 epochs and shows better plant leaf disease classification performance as well.

Moreover, we have used several activation functions in our model and got the best results with the swish function; hence, we have reported the results for the swish function only in the entire paper for the comparison of results. For better understanding, the comparative analysis with different activation functions is presented in Table 3. The basic reason that the proposed model performs well with the swish activation function is that the swish function is non-monotonic in nature. Due to this feature, it is possible for the output to still fall even if the input increases. This in return increases the information storage capacity of the proposed model and empowers it to learn a more discriminative set of underlying leaf patterns. While in comparison, the other activation functions lack this property; therefore, the model obtains better performance with the swish function in comparison with other activation functions.

**TABLE 3:** Comparative analysis of different activation functions.

Improved EfficientNetV2 Method with different activation function	Accuracy (%)
Sigmoid	99.89
ReLU	99.93
PReLU	99.94
LeakyReLU	99.96
Swish	99.99

### Evaluation of the Proposed Method

To build an accurate framework for plant disease classification, it must be capable of differentiating the various classes and recognizing the diseased region from the input samples. For this reason, we have conducted an experiment to analyze the classification performance of the proposed approach over the PlantVillage dataset. We have computed the Precision, Recall, and F1-score of the employed framework, as these metrics assist to determine the recognition performance of a model. We have performed two types of analysis, where in the first, we have shown the class-wise results, while in the second, we have reported the overall performance evaluation on the entire dataset.

**FIGURE 7:**
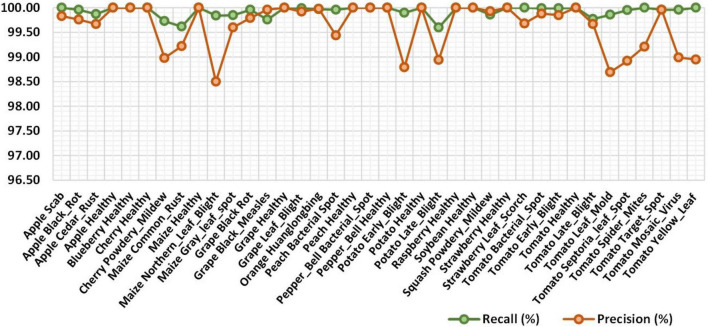
Class-wise precision and recall values of the proposed work.

**FIGURE 8:**
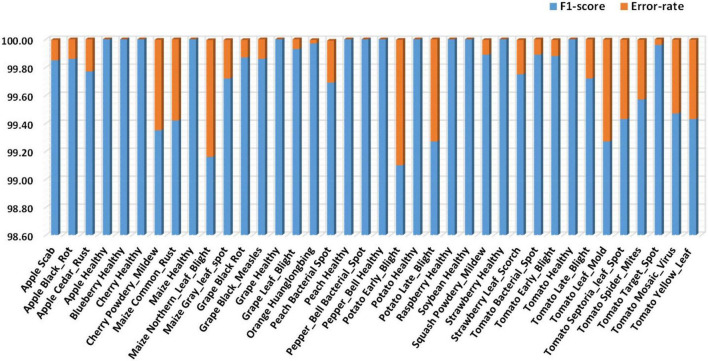
Class-wise F1-score and error rates.

For the first experiment, we have demonstrated the class-wise obtained precision and recall values in Figure 7, which is clearly showing that our approach can recognize all types of plant diseases with high performance. More specifically, we have obtained average precision and recall values of 99.63 and 99.93%, respectively. Moreover, the class-wise F1 score along with the error rate is reported in Figure 8. Figure 8 shows that our approach achieves the minimum and maximum error rate of 0–0.89% respectively. It is quite evident from Figures 7, 8 that our method effectively classifies the plant diseases and exhibits robust performance over various classes and on the entire dataset.

To further demonstrate the robustness of our framework, we have reported the classification performance of all 38 plant classes. More specifically, from class 1 to class 38, we have acquired the accuracy values of 99.84, 100, 100, 100, 100, 100, 100, 100, 100, 100, 99.80, 100, 100, 100, 100, 99.98, 100, 100, 100, 100, 100, 100, 100, 100, 100, 100, 100, 100, 100, 100, 100, 100, 100, 100, 100, 100, 100, and 100%, respectively. The improved EfficientNetV2 framework obtains the average accuracy value of 99.99%, which is clearly exhibiting the generalization ability of the proposed solution.

**FIGURE 9:**
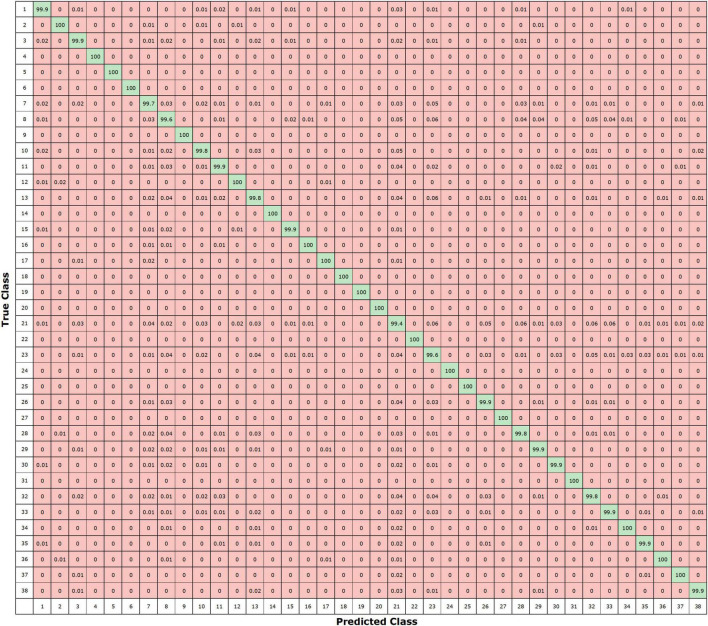
Confusion matrix of the presented method.

Moreover, we have shown the confusion matrix to additionally analyze the crop leaf disease categorization ability of the custom EfficientNetV2 network as the confusion matrix can better present the evaluation performance by showing the actual and predicted results. More clearly, we obtain the true positive rate (TPR) of 99.54, 100, 100, 100, 100, 100, 100, 100, 100, 100, 99.60, 100, 100, 100, 100, 99.98, 100, 100, 100, 100, 100, 100, 100, 100, 100, 100, 100, 100, 100, 100, 100, 100, 100, 100, 100, 100, 100, and 100%, for classes from 1 to 38, respectively. It is quite evident from the Figure 9 that the proposed modified EfficientNetV2 approach can accurately distinguish the leaf diseased portion of various classes and exhibits an improved recall rate.

It can be concluded from the performed result analysis that the presented improved EfficientNetV2 approach is efficient to distinguish and classify the images of varying classes. The main reason for the robust performance of the custom EfficientNetV2 is that it employs MBConv blocks having SE-based attention, which presents a more representative set of image features by eliminating the redundant data and reducing the model complexity which in turn makes the network prone to model overfitting problems.

### Comparison With Deep Learning-Based Methods

We designed an experiment to evaluate the proposed method against other DL-based methods. For this reason, we have chosen several recent techniques, namely, Inception V4 (Alom et al., 2020), VGG-16 (Alippi et al., 2018), ResNet-50 (Akiba et al., 2017), ResNet-101 (Too et al., 2019), ResNet-152 (Too et al., 2019), DenseNet-201 (Solano-Rojas et al., 2020), EfficientNet (Atila et al., 2021), and EfficientNetV2 (Tan and Le, 2021). To have a fair comparison, we have considered both the obtained classification results over the PlantVillage dataset and discussed the complexities of the networks as well.

First, we have discussed the model complexities in terms of total trainable numbers of network parameters along with the sample processing time taken by all comparative methods. The comparative values are discussed in Table 4, from which it is clear that the presented framework comprises fewer training parameters and takes less time than the peered methods to accomplish the task of classifying the various plant diseases. More specifically, the VGG-16 model comprises the largest number of network parameters, while in the case of processing time, the Inception V4 is the most expensive approach. However, in comparison, the improved EfficientNetV2 model comprises only 14.4 million parameters that are less than all the methods and takes a minimum processing time of 1,053 s, which is showing the effectiveness of the proposed solution. The competitive analysis in Table 4 is clearly showing that the proposed approach provides a lightweight solution to plant disease classification.

**TABLE 4:** Comparative analysis of proposed approach in terms of computational complexity with the base framework.

Method	Total trainable framework parameters (Million)	Processing time (ms)
Inception V4	41.2	4042
VGG-16	119.6	1051
ResNet-50	23.6	1583
ResNet-101	42.5	2766
ResNet-152	58.5	4366
DenseNet-201	20	2573
EfficientNet	19.4	1548
EfficientNetV2	15.2	1125
Improved EfficientNetV2	14.4	1053

**FIGURE 10:**
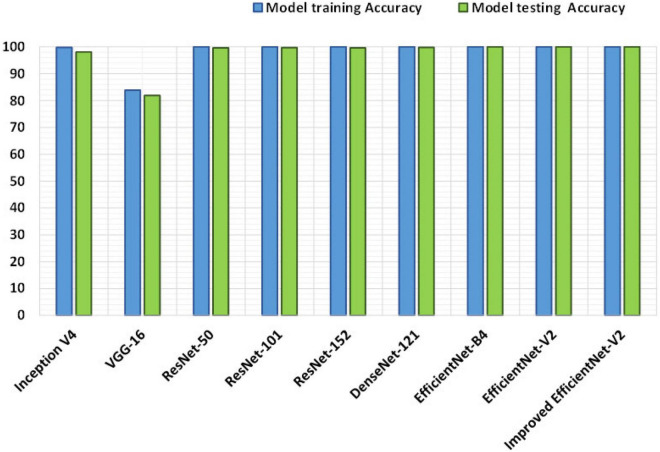
Performance comparison of the presented approach with the base methods.

Moreover, we have compared the classification accuracies of all selected DL-based approaches, and comparative analysis is shown with the help of bar graphs as given in Figure 10, as the bar graphs assist in better summarizing the large comparisons. More specifically, the approaches, namely, Inception V4, VGG-16, ResNet-50, ResNet-101, ResNet-152, DenseNet-201, EfficientNet, and EfficientNetV2 show the accuracy values of 98.08, 81.83, 99.59, 99.66, 99.59, 99.60, 99.97, and 99.98% respectively. However, the improved EfficientNetV2 model achieves an accuracy of 99.99% which is higher than all the competitive methods. In more detailed analyses, the peered methods show an average accuracy of 97.29%, so the custom EfficientNetV2 model gives an average performance gain of 2.7%.

The basic reason for the better plant disease classification performance of our approach is due to its shallow network structure which permits better employment of obtained information by eliminating the redundant data. Due to such a framework structure, the custom EfficientNetV2 minimizes the total model parameters in comparison to all approaches which contain very deep network architectures and suffer from the model over-fitting problem. These methods are not well generalized to all types of image post-processing attacks such as the presence of noise, blurring, and color variations in the suspected samples, suffering from high economical cost as well. Moreover, these methods do not perform well in real-world cases. The problems of comparative approaches are better resolved by the improved EfficientNetV2 model. As it contains a less deep framework structure with the power of better nominating the representative set of image deep features, which assists the EfficientNetV2 approach to enhance the classification performance while decreasing the computational cost as well. Furthermore, the proposed method is capable of detecting and classifying the image diseased region from the real-time captured samples *via* using drone technology. Therefore, it can be concluded that the improved EfficientNetV2 model provides both efficient and effective solutions to plant disease classification.

### Comparison With Machine Learning-Based Approaches

We have designed an analysis to evaluate the classification performance of our work against the ML-based classifiers using the PlantVillage database. To accomplish this task, we have considered many well-known ML classifiers, namely, RF, extreme learning machine (ELM), DT, SVM, and KNN, and comparative results are shown in Table 5. We have compared the techniques both in terms of classification accuracy and total trainable numbers of network parameters. It is depicted in Table 5 that the presented improved EfficientNetV2 method performs well than the ML-based classifiers with an accuracy of 99.99%. The SVM classifier attained the second-highest results with an accuracy of 98.01%, while the worst accuracy is shown by the DT classifier with a value of 77.8%. In more depth analysis, the comparative ML-based classifiers show an average accuracy value of 89.03%, which is 99.98% in our case. So, the presented custom EfficientNetV2 model gives an average performance gain of 10.96%. Moreover, we have compared the model parameters along with the execution time in Table 5. It is quite clear from the reported result that our method presents a lightweight solution to plant disease classification by showing the minimum model parameters and small execution time. It can be concluded from the obtained results that our model is more accurate for plant disease classification as compared with the ML-based classifier due to its better feature computation power which improves its recognition ability.

**TABLE 5:** Performance comparison of the presented framework with machine learning (ML)-based classifiers.

Classifier	Execution time (s)	Total trainable model parameters (Million)	Accuracy
Deep-keypoints along with the RF classifier (Ma et al., 2018)	–	–	93.4%
Deep-keypoints along with the ELM classifier (Xian and Ngadiran, 2021)	–	–	84.94%
Deep-keypoints along with the DT classifier (Xian and Ngadiran, 2021)	–	–	77.8%
Deep-keypoints along with the SVM classifier (Mohameth et al., 2020)	12 mn 21	25.5	98.01%
Deep-keypoints along with the KNN classifier (Mohameth et al., 2020)	12 mn 21	25.5	91.01%
Proposed	**17.5**	**14.4**	**99.99**%

*Bold means the architectures are improved.*

### Comparison With State-of-the-Art Techniques

In this section, a performance comparison of our technique, namely, the improved EfficientNetV2 model is conducted with several state-of-the-art approaches from the past employed for plant leaf disease categorization that used the PlantVillage database. We have evaluated the average results of comparative approaches as mentioned by Mohanty et al. (2016), Geetharamani and Pandian (2019), Mohameth et al. (2020), Sravan et al. (2021), Xiang et al. (2021) with our technique. Table 6 is exhibiting the comparison of our approach with the selected studies *via* using several standard evaluation metrics and by considering the total trainable network parameters as well. Sravan et al. (2021) proposed a DL-based method by employing the residual network for classifying the various crop leaf abnormalities. However, in the study by Xiang et al. (2021), a DL approach, namely, lightweight channel shuffle operation and multiple-size module (L-CSMS) was proposed to recognize several plant leaf diseases. The study by Mohanty et al. (2016) introduced a DL-based method, namely, GoogleNet, while in the study by Chen et al. (2020b) another framework, namely, MobileNet-Beta was introduced to automatically identify and categorize the plant leaf diseases. Geetharamani and Pandian (2019) presented a CNN approach to recognize and categorize the leaf diseases of several plants.

**TABLE 6:** Performance analysis of proposed method with recent techniques.

Method	Total trainable model parameters (Million)	Precision	Recall	F1-score	Accuracy
Residual Net (Sravan et al., 2021)	22	99.28%	99.26%	99.27%	99.26%
L-CSMS (Xiang et al., 2021)	5.44	–	–	–	97.90%
GoogleNet (Mohanty et al., 2016)	5	99.35%	99.35%	99.35%	99.35%
Custom CNN(Geetharamani and Pandian, 2019)	_	96.47%	99.89%	98.15%	96.46%
MobileNet-Beta (Chen et al., 2020b)	_	–	99.01%	–	99.85%
Proposed	**14.4**	**99.63%**	**99.93%**	**99.78%**	**99.99%**

*Bold means the architectures are improved.*

The reported results in Table 6 clearly show that the presented framework is more effective to plant leaf abnormality categorization as compared with the recent approaches. The improved EfficientNetV2 framework attained the average accuracy, precision, and recall values of 99.99, 99.63, and 99.93%, respectively, which are higher than all the comparative methods. More descriptively, the competitor methods show an average accuracy value of 98.56%, which is 99.99% in our case. So in the case of accuracy metric, the presented model gives an average performance gain of 1.43%, while in the case of precision, the comparative techniques show an average value of 98.37%, which is 99.63% for our work, so the custom EfficientNetV2 gives an average performance gain of 1.26%. Similarly, for the recall and F1-score, the presented work gives an average performance gain of 0.55 and 0.86%, respectively, as the methods in the studies by Mohanty et al. (2016), Geetharamani and Pandian (2019), Chen et al. (2020b), Sravan et al. (2021), Xiang et al. (2021) show an average recall and F1-score of 99.38 and 98.92%, which is 99.93 and 99.78% in our case, respectively. Therefore, the analysis depicts that our technique is more competent and proficient than the methods in the studies by Mohanty et al. (2016), Geetharamani and Pandian (2019), Chen et al. (2020b), Sravan et al. (2021)Xiang et al. (2021). Moreover, the studies by Mohanty et al. (2016), Xiang et al. (2021) show a small number of model parameters, however, at the expense of decreased model accuracy. The studies by Mohanty et al. (2016), Geetharamani and Pandian (2019), Chen et al. (2020b), Sravan et al. (2021), Xiang et al. (2021) have the model over-fitting problem due to their complex network structures, which in turn, increase the computational burden as well. While in comparison, the modified EfficientNetV2 approach presents a better tradeoff between the model parameters and classification performance. Our work uses the fused MBConv blocks which not only reduce the model training complexity but also assist to reuse the important computed image features which not only minimize the framework complication but also give it a computational advantage. Therefore, it can be concluded that our work gives a better solution to plant leaf disease classification.

## Conclusion

In this study, we have introduced a drone-based automated method, namely, improved EfficientNetV2 for the detection and classification of plant leaf diseases. More specifically, the customized EfficientNetV2 framework is employed as an end-to-end network to calculate the robust set of image keypoints and classify them in their respective classes. The proposed method can precisely recognize and categorize the various classes of plant leaf abnormalities from the PlantVillage database. Furthermore, the presented technique is proficient to crop disease categorization under the occurrence of several image distortions, i.e., changes in the brightness, contrast, color, position, angle, and structure of crop leaf diseases. The conducted result analysis clearly shows that our approach is more robust as compared with the recent techniques of crop plant leaf disease classification. Moreover, the modified EfficientNetV2 presents a low-cost solution to plant leaf disease classification which makes it effective for real-world scenarios. In the future, we plan to extend our work to other portions of plants such as stems and evaluate our work on more challenging databases.

## Data Availability Statement

The original contributions presented in this study are included in the article/supplementary material, further inquiries can be directed to the corresponding author/s.

## Author Contributions

WA: supervision, data curation, software, and writing—reviewing and editing. AJ: conceptualization, data curation, software, validation, and writing—reviewing and editing. MN: methodology, software, and writing—original draft preparation. MM: software, validation, and writing—reviewing and editing. SA: methodology, validation, writing—original draft preparation, and supervision. All authors contributed to the article and approved the submitted version.

## Conflict of Interest

The authors declare that the research was conducted in the absence of any commercial or financial relationships that could be construed as a potential conflict of interest.

## Publisher’s Note

All claims expressed in this article are solely those of the authors and do not necessarily represent those of their affiliated organizations, or those of the publisher, the editors and the reviewers. Any product that may be evaluated in this article, or claim that may be made by its manufacturer, is not guaranteed or endorsed by the publisher.
